# Deletion of RBPJK in Mesenchymal Stem Cells Enhances Osteogenic Activity by Up-Regulation of BMP Signaling

**DOI:** 10.1371/journal.pone.0135971

**Published:** 2015-08-18

**Authors:** Xifu Shang, Zhengliang Luo, Xudong Wang, Todd Jaeblon, John V. Marymont, Yufeng Dong

**Affiliations:** 1 Department of Orthopaedic Surgery, Anhui Provincial Hospital, Hefei, Anhui, China; 2 Department of Oral and Craniomaxillofacial Surgery, Ninth People's Hospital, Shanghai Jiaotong University School of Medicine, Shanghai, China; 3 Department of Orthopaedic Surgery, Louisiana State University Health Sciences Center, Shreveport, LA, United States of America; Second University of Naples, ITALY

## Abstract

Recently we have demonstrated the importance of RBPjk-dependent Notch signaling in the regulation of mesenchymal stem cell (MSC) differentiation during skeletogenesis both in vivo and in vitro. Here we further performed RBPJK loss-of-function experiments to demonstrate for the first time that RBPJK deficient MSC shows enhanced differentiation and osteogenesis acts via up-regulation of the BMP signaling. In the present study, we first compared the spontaneous and osteogenic differentiation in normal and recombination signal binding protein for immunoglobulin kappa J region (RBPJK) deficient human bone marrow-derived mesenchymal stem cells (MSCs). It was found that RBPJK highly expressed in fresh isolated MSCs and its expression was progressing down-regulated during spontaneous differentiation and even greater in osteogenic media inducted differentiation. Deletion of RBPJK in MSCs not only enhances cell spontaneous differentiation, but also significantly accelerates condition media inducted osteogenic differentiation by showing enhanced alkaline phosphatase (ALP) activity, Alizarin red staining, gene expression of Runx2, Osteopontin (OPN), Type I collagen (COL1a1) in culture. Additionally, BMP signaling responsive reporter activity and phosphor-smad1/5/8 expression were also significantly increased upon removal of RBPJK in MSCs. These data proved that inhibition of Notch signaling in MSCs promotes cell osteogenic differentiation by up-regulation of BMP signaling, and RBPJK deficient MSC maybe a better cell population for cell-based bone tissue engineering.

## Introduction

Mesenchymal stem cells (MSCs) hold promise for healing of injured tissue because of their capacity to differentiate into multiple cell lines and their availability from a wide variety of sources[1]. Although MSCs from different tissues, including dental pulp[2], adipose tissue[3], periosteum, circulating blood and the placenta have been shown to contribute to bone tissue regeneration in trauma and disease-based pre-clinical models [3–7], bone marrow derived MSC, one of the most widely studied stem cell population in the human body is still the most exciting cell source for skeletal tissue engineering by now.

Human bone marrow derived MSCs are well known not only by their anti-inflammatory and immuno-regulatory properties[8], but also by the capacity for multi-lineage differentiation into osteoblast, adipocyte, and chondrocyte and have great therapeutic potential for treating skeletal disease and facilitating skeletal repair [9–11]. Since the process from an undifferentiated MSC to a mature bone forming osteoblast contains multiple steps, so the time frame of MSC osteogenic differentiation is relatively long [12]. To identify factors that accelerate MSC differentiation towards osteoblastic cells becomes an important strategy for developing druggable targets for enhancing bone formation.

Various signaling factors have been implicated in the regulation of MSC osteoblastic differentiation, including Wnt[13], BMP[14], FGF[15] and PTH[16]. Recently, our group and others have identified the recombination signal binding protein for immunoglobulin kappa J region (RBPJK)-dependent Notch pathway as an important inducer of MSC proliferation, and an inhibitor of MSC differentiation during mouse limb-bud and postnatal bone development[17,18]. In humans and mice, Notch signaling is initiated when the Jagged 1 or 2 or Delta-like 1, 3, or 4 Notch ligands bind to cell surface Notch receptors (Notch1, Notch2, Notch3, or Notch4) on neighboring cells. This interaction induces cleavage and release of the Notch intracellular domain (NICD), which translocates from the cell surface into the nucleus to activate gene expression via a NICD-RBPJK-MAML transcriptional complex [18,19]. Although mouse genetic studies have demonstrated that conditional ablation of the Notch pathway components RBPJK in early MSCs of the limbs resulted in enhanced chondrogenic and osteogenic differentiation [17], but the underlying mechanism is still unknown. To further confirm inhibition of the Notch pathway in culture could promote the osteoblastic differentiation in human MSCs, here we aimed to investigate the following: 1) human MSC osteogenic differentiation following removal of RBPJK via shRNA transduction. 2) Cellular signaling changes following inhibition of the Notch pathway by deletion of RBPJK in culture.

## Materials and Methods

### Ethics Statement and Donor Data

Human bone marrow aspirate was harvested from the iliac crest of donors (n = 6) for MSC isolation. The mean age of the donors was 55 years (range 47–65) comprised four males and two females with no history or evidence of genetic disease or malignancy. The ethics committee of Anhui Provincial Hospital approved the use of the tissue material for this study (Approval No. 4353). Written informed consent was obtained from all donors and kept in locked file box since these patients were in spine fusion surgery using their own bone marrow aspirate.

### MSC isolation and culture

Method for isolation of human MSC based on red blood cell (RBC) lysis with ammonium chloride[20]. After incubation in RBC lysis buffer for 10 min, total bone marrow cells were seeded at 5 × 10^6^ cells/cm^2^ in alpha-minimum essential medium (α-MEM) supplemented with 15% FBS, 2 mM L-glutamine, 0.5% antibiotic/antimycotic solution (all from Gibco-BRL, Life Technologies) for 3 days. Cells were then washed 3–5 times with 1XPBS and incubated at 37°C in a humidified 95% air and 5% CO2 atmosphere, cultured up to 80% confluence, and then trypsinized (trypsin-EDTA solution, Gibco-BRL), centrifuged, and re-plated at a density of 2000 cells/cm^2^ for subsequent expansion. All experiments were performed with cells after the second or the third passage around 2 weeks.

### Flow cytometry analysis

MSC phenotype was evaluated after 2 weeks’ expansion by the expression of CD34-APC (BD Biosciences Pharmingen, San Diego, CA), CD45-FITC (BD Biosciences Pharmingen, San Diego, CA), CD90–PE (Dako Cytomation Denmark A/S, Glostrup, Denmark), CD73-PE (BD Biosciences Pharmingen) and CD105-FITC (RD Systems, Minneapolis, MN). Cells were incubated for 30 min at room temperature with antibodies and flow cytometry was performed on a LSR-II flow cytometer (Beckton Dickson). Isotype matched controls were included for each antibody and used to set the electronic gates on the flow cytometer. The data were analyzed using FlowJo software (Tree Star).

### Lentivirus-mediated shRNA cell infection

RBPJK-specific (shRBPJK) and control (shCo) short hairpin RNA (shRNA) lentiviral particles were prepared by GeneChem (Shanghai, China, http://www.genechem.com.cn). For lentiviral infection, 2,000 cell/mm^2^ MSCs were seeded in 6-well plates and incubated for 24 h at 37°C prior to be added with shRNA lentivirus condition medium in the presence of 8 μg/ml polybrene (Sigma-Aldrich). The infected MSC cultures were harvested at various time points (2, 3, and 14 days). RBPJK expression in cells was detected by Real time RT-PCR and Western blot analysis.

### BrdU labeling

Cell proliferation assays were performed using a BrdU ELISA Kit (Roche). Briefly, shRNA lentiviral infected MSCs were cultured at 2000 cells/cm^2^ for 2 days, then exposed to BrdU labeling reagent for 6 hours followed by incubation with FixDenat buffer for 30 min and detection with anti-BrdU-POD working solution. Absorbance values were measured by a multi-mode microplate reader (BioTek Instruments) at 450 nm.

### MSC differentiation assay

MSC spontaneous differentiation assays were performed using standard stem cell growth media. MSCs were cultured at 2,000 cells/cm^2^ to confluence on standard culture dishes, growth media was changed every 3 days till harvesting at day 3 and 14. RNA isolation and ALP staining were performed as previously described [17]. For cell osteogenic differentiation assay, MSCs were cultured at 2,000 cells/cm^2^ to confluence on standard culture dishes. Stem cell growth media were then replaced with osteogenic induction media supplied with the Differentiation Media BulletKit—Osteogenic (Lonza). RNA isolation and Alizarin red staining were performed at 3 and 14 days of culture as previously described [21].

### Transfection and luciferase assay

The MSC cells were prepared in 12-well plates in triplicate and co-transfected with BMP/Smad responsive element-luciferase reporter (12XSBE) plasmids (500 ng/well) by utilizing Lipofectamine 2000 reagent (Invitrogen, Carlsbad, CA) prior to be added shRBPJK lentiviral condition medium and BMP-2 (50 ng/ml). After 48-h incubation, the cells were washed twice with ice-cold PBS and harvested with a reporter lysis buffer (Promega, Madison, WI). SV40 was co-transfected as internal control. The luciferase activity was analyzed using the dual-luciferase assay system (Promega, Madison, WI) as described previously [22].

### Real time RT-PCR

cDNA was synthesized from 1 μg total RNA using the SuperScript III reverse transcriptase kit (Invitrogen) in a final volume of 20 μl. Primers were designed with the IDT SCI primer design tool (Integrated DNA Technologies, San Diego, California). RT-PCR experiments were performed with a Bio-Rad C1000 thermal cycler (Bio-Rad, Hercules, CA), and real-time PCR experiments were performed with an ABI prism 7500 (Applied Biosystems, Grand Island, NY) in triplicate. Sequence for each primer pair were: RBPJK, forward primer 5′- GAGCGAGGGGATCAAACAGT-3′, reverse primer 5′-GCTGCTGCATTTCTTGGTCA-3′; Alkaline phosphatase (ALP), forward primer 5′-GGGCATTGTGACTACCACTC-3′, reverse primer 5′-AGTCAGGTTGTT CCGATTCA-3′; Runx2, forward primer 5′-CACTGCCACCTCTGACTTCT-3′, reverse primer 5′-CACCATCATTCTGGTTAGGC-3′; Osterix (OSX), forward primer 5′-TGGCCATGCTGACTGCAGCC-3′, reverse primer 5′-TGGGTAGGCGTCCCCCATGG-3′; Osteopontin (OPN), forward primer 5′-AAGGAACCAAAGCATCAAGAATTAG-3′, reverse primer 5′-AGATGTCATCAGGCAGCTTGAC-3′; Type I collagen (COL1a1), forward primer 5′-GTTTGGCCTGAAGCAGAGAC-3′, reverse primer 5′-TCTAAATGGGCCACTTCCAC-3′; β-actin, forward primer 5′-ACCACAGTCCATGCCATCAC-3′; reverse primer 5′-TCCACCACCC TGTTGCTGTA-3′. Samples were analyzed in triplicate and the raw data consisted of PCR cycle numbers required to reach a fluorescence threshold level. The relative expression level of target genes was normalized with geNorm software (Primer Design Ltd) using β-actin gene as reference to determine the normalization factor [23].

### Alizarin red staining

To measure extracellular matrix Ca deposits for bone nodule formation, cellular matrix was stained using Alizarin red dye, which combines with Ca in the matrix as previously described [24]. At day 14 following the induction with osteogenic condition media, cells were washed with PBS twice and then fixed with 2.0% formaldehyde. The cells were stained with 40 mmol/L of Alizarin red solution (pH 4.4) for 40 mins at room temperature and rinsed with deionized water twice. The images of stained cells were captured using a phase contrast microscope with a digital camera (IM50, Leica, Germany). Quantification of Alizarin red staining was performed as previously described [25], 1.5 ml of 50% acetic acid was added to stained plates for 18 hours at room temperature to ensure that all of the bound dye was dissolved. After the 18 hours de-stain, 500 μl of the solubilized stain was pipetted into a 1.5 ml tube containing 600 μl of 1 M NaOH in order to adjust the pH to 4.1. 100 μl of this solution was then transferred to a 96-well plate and absorbance measured at 550 nm using a multi-mode microplate reader (BioTek Instruments). Absorbance values were compared to each other to determine any change in mineral content as a result of mineralization.

### Western blot analysis

MSCs were lysed in RIPA buffer (10 mM Tris–HCl, 1 mM EDTA, 1% sodium dodecyl sulfate [SDS], 1% NP-40, 1: 100 proteinase inhibitor cocktail, 50 mM β-glycerophosphate, 50 mM sodium fluoride). The samples were separated on a 10% SDS polyacrylamide gel and transferred to polyvinylidene difluoride (PVDF) membranes with a semi-dry transfer apparatus (Bio-Rad). The membranes were blotted with 5% dehydrated milk for 2 h and then incubated with primary antibodies overnight. The immune complexes were incubated with horseradish peroxidase-conjugated anti-rabbit or anti-mouse IgG (Promega, USA) and visualized with SuperSignal reagents (Pierce, USA). Primary polyclonal antibodies against RBPJK, phosphor-smad1/5/8, phospho-ERK1/2, phospho-p38MAPK, total-ERK1/2 and total-p38MAPK (Cell Signaling, USA) were used. We also used a primary monoclonal antibody to detect the housekeeping protein, β-actin (Sigma-Aldrich, USA).

### Statistical Analysis

All experiments were repeated at least three times independently. All data were presented as mean ± s.d. Statistical significance among the groups was assessed with one-way ANOVA. The level of significance was p < 0.05.

## Results

### Human MSC isolation and characterization

**Fig 1:**
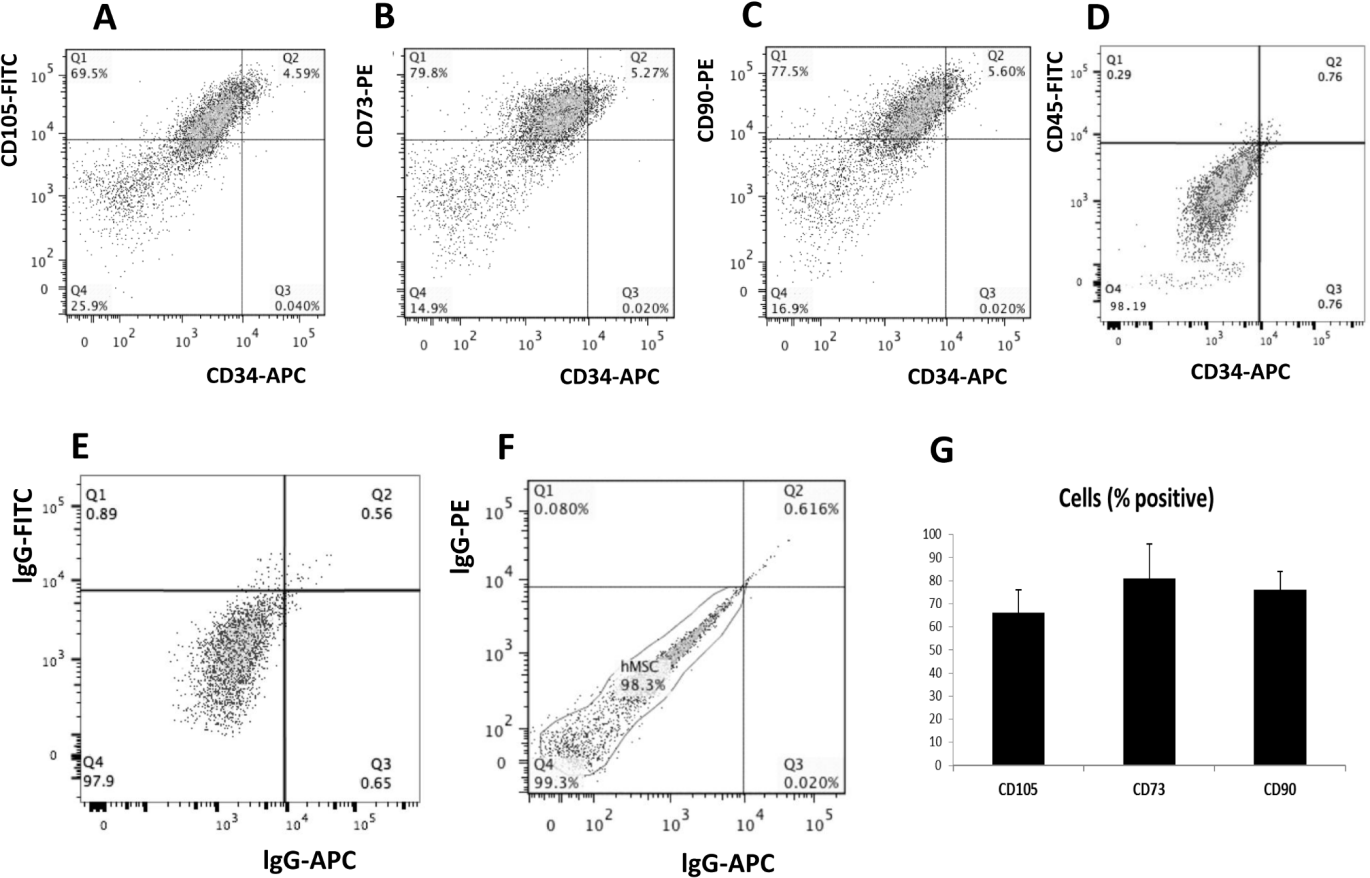
Human MSC isolation and characterization. Newly isolated human Mesenchymal stem cells (MSCs) were cultured in stem cell growth media for up to 80% confluence before being harvested for Flow Cytometry. (A) Representative Flow Cytometry histograms showing CD105 expression in 6 different isolations of MSCs. (B) Representative Flow Cytometry histograms showing CD73 expression in 6 different isolations of MSCs. (C) Representative Flow Cytometry histograms showing CD90 expression in 6 different isolations of MSCs. (D) Representative Flow Cytometry histograms showing CD45 and CD34 in 6 different isolations of MSCs. (E, F) Representative Flow Cytometry histograms showing isotype controls, FITC versus APC and PE versus APC, in 6 different isolations of MSCs. (G) Quantification of the CD105, CD90, CD73 subpopulations in total MSCs.

To isolate functional stem cells is important not only to study the molecular mechanisms but also for the establishment of stem cell–based therapeutics. Here we adopted a protocol to isolate human bone marrow derived MSC using plastic adherent method, as previously described [20]. After 2 weeks expansion, cells were harvested for flow cytometry analysis and further experiments. Flow cytometry analyses demonstrated that cells obtained from six separate preparations ranged from 69.5% to 79.8% positive for stem cell markers CD105 (Fig 1A), CD73 (Fig 1B) and CD90 (Fig 1C). To eliminate lymphocytes and leukocytes contamination, lymphocyte marker CD45 and hematopoietic marker CD34 were further analyzed, Fig 1D showed both CD45 and CD34 positive populations are less than 0.5% in newly isolated human MSCs confirmed that our MSCs is not contaminated with lymphocytes and hematopoietic stem cells. As negative controls, the appropriately matched isotype controls, including lgG-FITC, lgG-PE and lgG-APC, were utilized for each antibody to set the gate for positive population (Fig 1E and 1F). Finally, the average expression of three positive MSC cell surface markers from all isolations was analyzed and shown in Fig 1G. These data suggest that our MSC preparations are enriched with cells expressing common MSC cell surface markers, and therefore they were utilized for subsequent experiments.

### Deletion of RBPJK in MSC enhances cell differentiation

**Fig 2:**
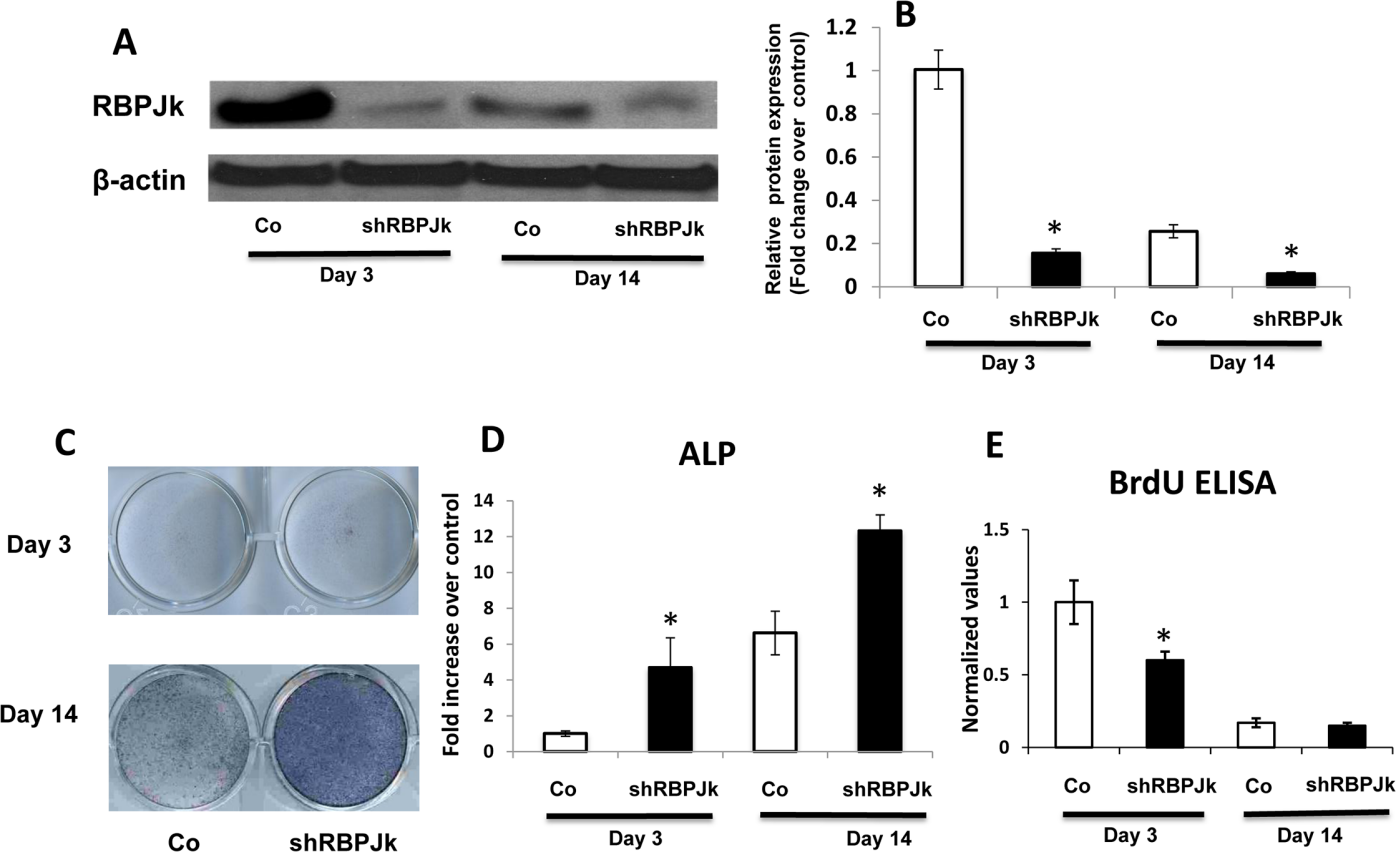
Deletion of RBPJK enhances cell spontaneous differentiation in regular MSC culture. Mesenchymal stem cells were infected with Control shRNA(Co) or RBPJK shRNA (shRBPJK) lentivirus before being harvested for alkaline phosphatase (ALP) staining, western blot, RT-PCR analysis and BrdU ELISA. (A) Western Blot showed a significant decrease of RBPJK protein expression at day 3 and 14 following shRBPJK lentiviral infection. (B) Fold change in protein level of RBPJK in Western blots was determined by measuring band intensity with ImageJ. (C) Deletion of RBPJK resulted in increased ALP staining at days 3 and 14, compared to controls (Co). Scale bars, 100μm. (D) ALP gene expression was increased at days 3 and 14, with maximal increase at day 14 in RT-PCR analysis. (E) BrdU ELISA to monitor both MSCs proliferation at days 3 and 14 after lentiviral infection. Data are means ± s.d. of three independent experiments performed in duplicate and the control gene expression level at day 3 was set at 1. (*, *p* < 0.05 compared with control at same time point).

To determine the importance of RBPJK during MSC maintenance and differentiation, a loss of function experiment was performed. In this experiment, lentivirus that contains specific shRNA for RBPJK was used to knock down RBPJK in MSCs. To confirm RBPJK expression was successfully inhibited in MSCs, RNA and protein from control MSCs (shCo/MSCs) and RBPJK deficient MSCs (shRBPJK/MSCs) were analyzed by PCR and western blot. Fig 2A showed there was no detectable RBPJK expression in RBPJK shRNA lentiviral infected MSCs by Western blotting analysis when compared to shCo/MSCs at day 3 after infection. Although we saw a mild detectable expression of RBPJK at day 14 in lentiviral infected MSCs, and the level is significant lower than that in shCo/MSCs. These fold changes in protein level of RBPJK in Western blots were further quantified by measuring band intensity (Fig 2B).

To examine the effects of an absence of RBPJK on MSC spontaneous differentiation, the shRBPJK/MSCs were cultured in stem cell growth medium for up to 14 days. Compared with shCo/MSCs, spontaneous differentiation was dramatically accelerated in shRBPJK/MSCs from day 3 to day 14 (Fig 2C), according to the enhanced ALP staining in shRBPJK/MSCs. This enhanced ALP expression was further confirmed by real time PCR data (Fig 2D). Since the spontaneous differentiation of shRBPJK/MSCs was significantly increased, we speculate that RBPJK may function as a differentiation repressor in MSCs because of its ability to maintain MSC in un-differentiation stage.

To determine whether Notch inhibition by deletion of RBPJK further regulates proliferation of MSCs. We performed BrdU ELISA assays using day 3 and day 14 MSCs infected with shRBPJK lentiviral particles. Our data demonstrate that deletion of RBPJK decreases BrdU incorporation by more than 40% as compared to control MSCs at day 3, but no significant difference was observed at day 14 when MSCs reached more than 100% confluence in both groups (Fig 2E). Taken together, these data support the concept that block Notch signaling by deletion of RBPJK promotes cell differentiation and inhibits cell proliferation in MSC culture.

### Deletion of RBPJK enhances osteogenic media inducted osteogenesis

**Fig 3:**
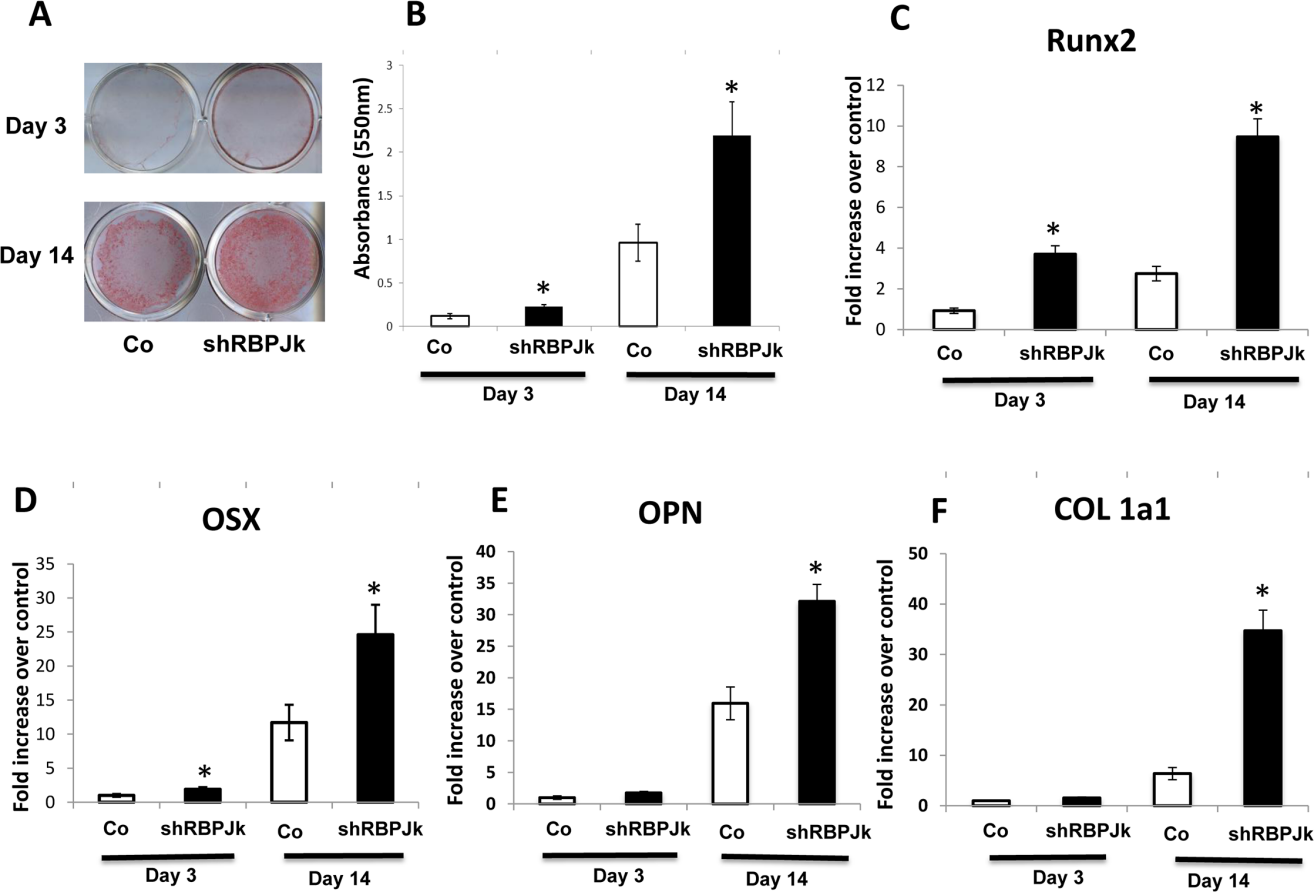
Inhibition of Notch signaling by deletion of RBPJK enhances inducted MSCs osteogenic differentiation. (A) An increase in osteogenic nodule formation was observed in shRBPJK lentivirus infected MSCs at both day 3 and day 14, with maximal staining of Alizarin red at day 14. Scale bars, 100 μm. (B) Quantification of Alizarin Red Staining indicates a significant increase (p < 0.05) in mineral content between lentivirus control (Co) and shRBPJK lentivirus infected MSCs. (C, D, E, F) Runx2, Osterix (OSX), Osteopontin (OPN), and type I collagen (COL1a1) gene expression were significantly increased in RBPJK deficient MSCs at day 14. Data are means ± s.d. of three independent experiments performed in duplicate and the shRNA-lentivirus control (Co) gene expression level was set at 1. (*, p < 0.05 compared with control at day 3)

To test if shRBPJK infected MSC could be rapidly induced to osteoblasts, osteoblastic differentiation assay was performed using both shCo/MSCs and shRBPJK/MSCs. Monolayer MSCs were cultured in osteogenic-induction media for up to 14 days and Alizarin Red staining was used to characterize the biological mineralization of differentiated osteoblast. Compared with shCo/MSCs, Alizarin red staining was dramatically enhanced in shRBPJK/MSCs at day 3 and day 14, with a maximal increase at day 14 (Fig 3A). Quantification of Alizarin Red Staining indicates a significant increase (p < 0.05) of mineral content in shRBPJK/MSCs when compared to shCo/MSCs (Fig 3B). MSC to an osteoblast occurs in different phases, and each of these phases is characterized by a particular pattern of expressed osteoblast marker genes. Since Runx2 is best known as the master regulator of osteoblast differentiation and osteoblast marker gene expression as well as osteoblast function, we further measured expression of Runx2 and OSX, a transcription factor that is directly regulated by Runx2 during MSC osteogenic differentiation. Real time PCR data demonstrate that Runx2 expression was increased from day 3 to day 14 in both shCo/MSCs and shRBPJK/MSCs. More importantly, Runx2 level was significantly higher in shRBPJK/MSCs than that in shCo/MSCs at days 3 and 14 (Fig 3C). Similar RNA expression pattern of OSX was also observed at both time points (Fig 3D). Additionally, expression of OPN and COL1a1, two mature osteoblast markers, were increased by 15-fold and 7-fold respectively at day 14 when compared to day 3 in shCo/MSCs. Interestingly, these increases were further potentiated when RBPJK was deleted from MSCs at day 14 (Fig 3E and 3F).

### Deletion RBPJK in MSC enhances BMP signaling

Next, we performed experiments to delineate the underlying mechanism for the enhanced osteogenic ability in shRBPJK/MSCs. Since BMP signaling affects all aspects of skeletal development and regeneration [15,26], we then analyzed BMP signaling response upon the immediate loss of RBPJK in MSCs.

**Fig 4:**
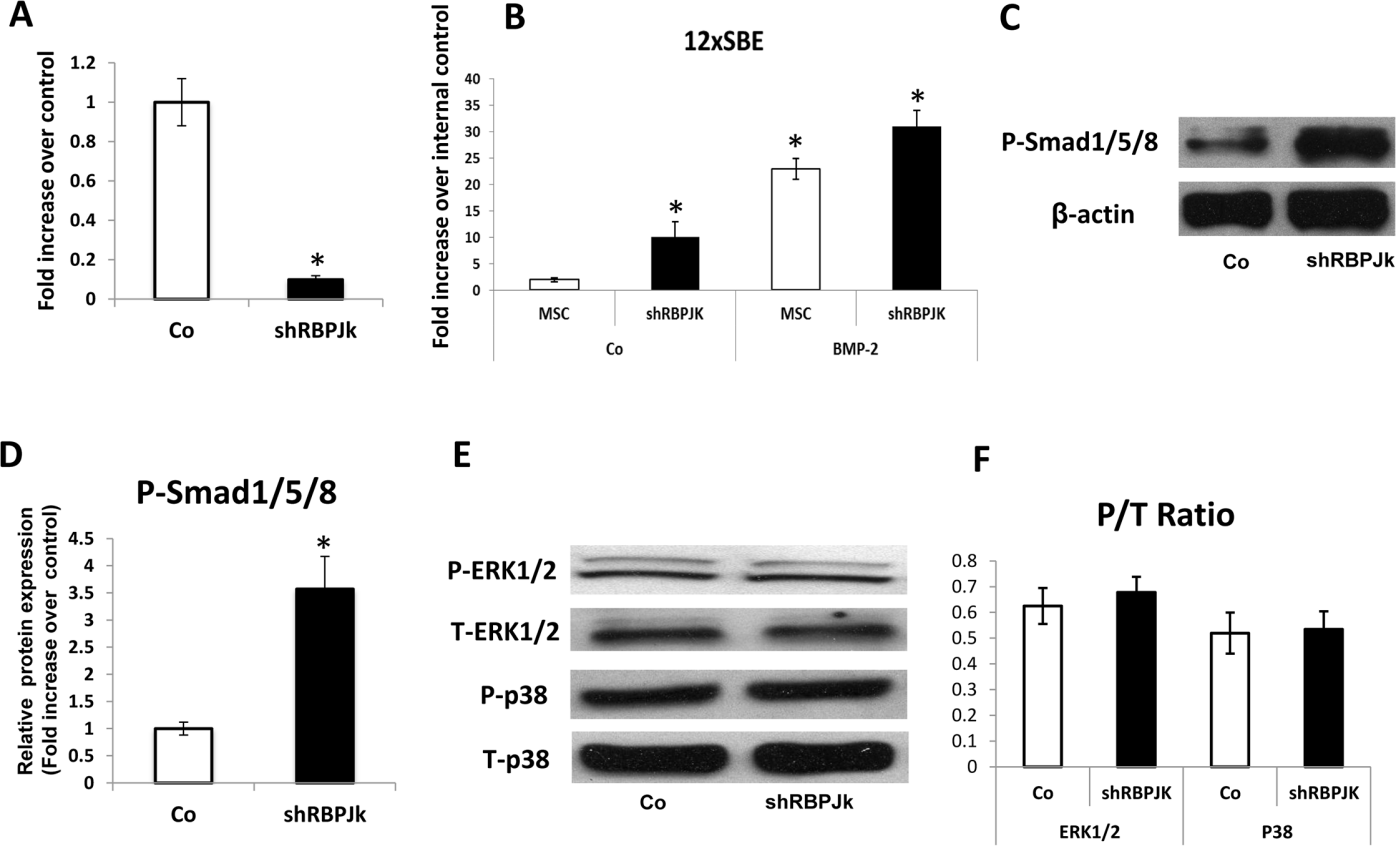
Enhanced BMP signaling in RBPJK deficient MSCs. (A) Real time RT-PCR analysis reveals that expression of RBPJK was significantly inhibited by 90% in shRBPJK lentivirus infected MSCs (shRBPJK) at day 2 when compared to shRNA-lentivirus control MSCs (Co). (B) Luciferase assays showed a significant increase of BMP responsive reporter activity in shRBPJK MSCs and this transcriptional up-regulation is further enhanced by BMP-2 treatment. Data are means ± s.d. of three independent experiments performed in duplicate and all the results were normalized to internal control (*, p < 0.05 compared with control MSCs (Co) without BMP-2 treatment). (C) Western Blot shows phosphor- smad1/5/8 (P-smad1/5/8) protein levels were significantly enhanced by deletion of RBPJK in MSC culture at day 2. β-actin was used as a loading control. (D) Quantification of P-smad1/5/8 protein level in Western blots was determined by measuring band intensity with ImageJ. (E) Western Blot shows deletion of RBPJK did not cause a change in phosphor-ERK1/2 (P-ERK1/2), total-ERK1/2 (T-ERK1/2), phosphor-p38 (P-p38) and total-p38 (T-p38) protein levels in MSC culture at day 2. (F) Densitometry quantification of activated P-ERK1/2 and P-p38, expressed as a ratio to total ERK and total p38 by P/T ratio. Data are means ± s.d. of three independent experiments. (*, p < 0.05 compared with shRNA-lentivirus control MSCs).

To determine whether RBPJK deletion in MSCs might affect BMP signaling during differentiation, we first transfected MSCs with BMP responsive reporter 12XSBE. After 4 h of incubation, shRBPJK lentivirus and BMP-2 were then added to the medium to knock down RBPJK expression and stimulate BMP signaling. Fig 4A showed that 90% deletion of RBPJK was reached after 2 days of infection. More importantly, without treatment of BMP-2, shRBPJK/MSCs showed a 9-fold increase of BMP reporter activity compared to shCo/MSCs, and this increase was further potentiated by BMP-2 treatment (Fig 4B). Moreover, western blot showed a significant increase of phospho-Smad1/5/8 expression in shRBPJK/MSCs as compared with shCo/MSCs (Fig 4C and 4D) indicating Smad-dependent BMP signaling was indeed activated in cells. To further determine whether RBPJK deletion could be affected by Smad-independent pathway [27,28], expression of phospho-ERK1/2, phospho-p38MAPK, total-ERK1/2 and total-p38MAPK was measured at protein level. Western blot results in Fig 4E showed no significant difference in level of phospho-ERK1/2, phospho-p38MAPK, total-ERK1/2 and total-p38MAPK was observed between shRBPJK/MSCs and shCo/MSCs (Fig 4E). This no difference result was further confirmed by quantification of protein bands in Western Blot by ImageJ (Fig 4F). Therefore, deletion of RBPJK enhances MSC osteogenic differentiation likely in part through promoting intracellular Smad-dependent BMP signaling.

## Discussion

A number of in vitro and in vivo studies suggest osteoblastic differentiation of MSCs is associated with several lineage-specific transcription factors, including smad1/5/8, β-catenin, core-binding factor 1 (CBFA1/Runx2), and Osterix (OSX) [29],[30]. Additionally, some growth factors, hormones and extra-cellular matrix components, such as BMP, PTH are also known to induce MSC osteoblastic lineage specific differentiation. Here we demonstrate that Notch signaling transcriptional factor RBPJK not only expressed in MSCs, but also plays an important role in MSC maintenance. Our gene knock-down experiments clearly showed that RBPJK deficient MSCs started to express higher level of ALP at day 3 and day 14 in culture when compared to shCo/MSCs suggest an accelerated spontaneous differentiation in cultured RBPJK deficient cells. This result is consistent with our previous finding that conditional ablation of the Notch pathway components (Notch 1 and 2, RBPJK) in early MSCs of the limbs resulted in enhanced chondrogenic and osteogenic differentiation followed by a depletion of the MSC pool in vivo [17], and further indicates that the role of RBPJK in MSCs is to maintain stem cell “stemness” and prevent cell differentiation.

Previous study demonstrates a RBPJK-dependent regulation of bone formation in vivo, in that deletion of RBPJK in limb bud progenitor cells leads transit increases bone mass [17]. We then asked whether RBPJK deficient MSCs are a more readable MSC population for osteogenesis. Results from our osteogenic differentiation assay clearly showed an enhanced bone mineralization in RBPJK deficient MSCs. This accelerated bone cell formation was further confirmed by increased expression of Runx2, which controls expression of osteoblast marker genes by binding to transcription factor, Osterix [30]. Since Runx2 binding site was also found in the promoter region of all major osteoblast marker genes, including OPN and COL1a1, we further measured gene expression of OPN and COL1a1 in RBPJK deficient MScs, and a similar expression pattern to Runx2 was observed for both OPN and COL1a1, suggest inhibition of Notch signaling by deletion of RBPJK may promote MSC osteogenic differentiation through up-regulation of Runx2 expression.

Given the crucial roles of BMP signaling in bone tissue developmental processes and Runx2 is one of the target gene induced by BMP signaling, it will be important to investigate the interaction between RBPJK and BMP signaling components during differentiation of MSCs. Here RBPJK-deficient MSCs exhibited an increased BMP signaling reporter activity and enhanced expression of phosphor-smad1/5/8 when compared with the controls, suggest that RBPJK deletion may promote MSC differentiation towards osteoblast by inducing Runx2 through upregulation of BMP signaling. In addition to the canonical Smad-dependent signaling pathway, mitogen-activated protein kinases (MAPKs) such as p38 or ERK1/2 can also be activated by BMPs via Smad-independent mechanisms. To eliminate the possible involvement of MAPK signaling in RBPJK deletion induced cell differentiation, phosphorylated active ERK1/2 and p38 were further detected by Western Blot. Our results clearly showed deletion of RBPJK did not induce activation of the MAPK signaling and demonstrated that RBPJK activity in MSCs can be attributed only to its BMP-activated Smad signaling pathway.

## Conclusion

In conclusion, our study provides novel and important insights into the role of RBPJK during early MSC maintenance and differentiation. We demonstrate for the first time that deletion of RBPJK in MSCs effectively enhances the cell osteogenic differentiation in vitro likely in part by co-activating Smad-dependent BMP signaling. Since RBPJK deficient MSC is a better MSC population for bone tissue engineering, identification of factors could be used to transient suppression of RBPJK expression to enhance the MSC osteoblastic differentiation during bone tissue regeneration become one of our future research focuses.
